# HEMATOPOIETIC LOSS OF THE Y CHROMOSOME AND AGING PHENOTYPES

**DOI:** 10.1093/geroni/igae098.1449

**Published:** 2024-12-31

**Authors:** Nick Chavkin

**Affiliations:** Seattle Children’s Research Institute & University of Washington, Seattle, Washington, United States

## Abstract

Life expectancy of men is six years less than women in developed countries. Recent epidemiological studies suggest that a significant portion of this discrepancy can be attributed to an age-related phenomenon in blood. As men age, a portion of their blood cells will lose the Y chromosome. This phenotype, termed Mosaic Loss of the Y Chromosome (mLOY), is the most common post-zygotic mutation in humans and is present in almost half of men by age 70. mLOY has been correlated with increased mortality and many age-related diseases. Recently, we have found that men with higher mLOY are more susceptible to non-ischemic heart failure. Using a mouse model of mLOY, we showed causal effects of mLOY on diminished lifespan, increased progression of age-associated cardiomyopathy, and increased progression of cardiac fibrosis after injury. Further analysis revealed that mLOY macrophages showed both an increased propensity towards pro-fibrotic macrophage polarization and increased expression of pro-fibrotic ligands. Genetic analysis of the Y chromosome identified the gene UTY as a potential causal locus for mLOY-mediated effects on cardiac fibrosis. Finally, analysis of published single cell RNA sequencing datasets from human male heart, lung, and kidney tissue suggests a broad correlation between mLOY and fibrotic pathology with potential direct effects of mLOY macrophages on tissue-resident fibroblasts. These mechanistic findings help explain the correlation between mLOY and cardiac fibrosis, and further suggest general effects of mLOY on age-related phenotypes.

